# Trauma and its consequences in Iran: cross-cultural adaption and validation of the Global Psychotrauma Screen in a representative sample

**DOI:** 10.1186/s12888-023-04564-8

**Published:** 2023-01-25

**Authors:** Yahya Salimi, C. Hoeboer, Seyed Ali Motevalli Haghi, R. E. Williamson, Mohammad Dawood Rahimi, Nader Rajabi-Gilan, Ali Almasi, M. Olff

**Affiliations:** 1 Social Development & Health Promotion Research Center, Health Institute, Kermanshah University of Medical Sciences, Kermanshah, Iran; 2 Department of Psychiatry, Amsterdam Public Health, Amsterdam UMC Location University of Amsterdam, Meibergdreef 9, Amsterdam, Netherlands; 3 Cognitive Neuroscience and Clinical Neuropsychology, Department of General Psychology, University of Padova, Padua, Italy; 4 Department of Psychology, University of Montana, Missoula, MT USA; 5 Cognitive Psychology, Ferdowsi University of Mashhad, Mashhad, Iran; 6 Sociology Department, Faculty of Humanities and Social Sciences, University of Kurdistan, Sanandaj, Iran

**Keywords:** Screening, Psychotrauma, PTSD, Complex PTSD, Anxiety, Depression

## Abstract

**Background:**

Potentially traumatic events may lead to the development of a wide range of adverse psychological responses, including symptoms of anxiety, depression, and (complex) posttraumatic stress disorder (PTSD). Despite the high prevalence of potentially traumatic events in Iran, there is no population data nor evidence-based instrument to screen for cross-diagnostic psychological responses to trauma. The Global Psychotrauma Screen (GPS) is a transdiagnostic self-report instrument for the detection of trauma-related symptoms, as well as risk and protective factors related to the impact of potentially traumatic events.

**Objective:**

The present study seeks to 1) translate and cross-culturally adapt the GPS in the Persian (Farsi) language and 2) examine the psychometric properties of the Persian GPS.

**Method:**

The translation and adaptation were performed using the Sousa and Rojjanasrirat (2011) method. A pilot study (*n* = 30) was carried out to test the content validity and test–retest reliability of the GPS. Next, in a representative sample (*n* = 800) of residents of Kermanshah City, the GPS, the General Health Questionnaire (GHQ) and the PTSD Checklist for DSM-5 (PCL-5) were administered. Construct validity of the Persian GPS was assessed using exploratory and confirmatory factor analysis. Additionally, we evaluated the convergent validity and internal consistency of the GPS.

**Results:**

Exploratory and confirmatory factor analyses indicated a three-factor model as the best solution with factors representing 1) Negative Affect, 2) Core PTSD symptoms and 3) Dissociative symptoms. The GPS total symptom score had high internal consistency and high convergent validity with related measures. A GPS total symptom cut-off score of nine was optimal for indicating a probable PTSD diagnosis based on the PCL-5. About half (52%) of the current sample met criteria for probable PTSD.

**Conclusions:**

The current findings suggest that the GPS can be effectively adapted for use in a non-Western society and, specifically, that the Persian GPS represents a useful, reliable and valid tool for screening of trauma-related symptoms in Iran.

**Supplementary Information:**

The online version contains supplementary material available at 10.1186/s12888-023-04564-8.

## Introduction

Trauma is a global issue and a public health concern [1–3]. In Iran, potentially traumatic events are prevalent, including sexual assaults, wars, earthquakes, floods and other natural disasters [4–8]. Kermanshah city, located in the western part of Iran, has experienced multiple wars over the past eight years, involving both airstrikes and ground battles [9]. The area is also one of the most earthquake-prone areas of the world, with recent earthquakes in 2017 and 2018 resulting in more than 600 casualties [10]. The recent COVID-19 pandemic has further added to the stress burden within the country.

Potentially traumatic events may elicit stress reactions and lead to psychological disorders such as posttraumatic stress disorder (PTSD) and depression. These disorders are related to functional impairments, lower quality of life, work-related problems and physical health problems [11–14]. Accurate and easily administered assessment of these disorders is important to identify those in need of treatment. Although several instruments exist in Iran, such as the Impact of Event Scale Revised (IES-R) [15], Watson Post Traumatic Stress Disorder (PTSD) interview [16], PTSD Checklist, military edition (PCL-M) [17], PTSD Checklist for DSM-5 (PCL-5) [18] and National Stressful Events Survey as an updated scale [19], there is no brief screening tool available that assesses the wide range of psychological reactions to a potentially traumatic event.

The Global Psychotrauma Screen (GPS) is a brief self-report instrument that screens for a range of trauma-related psychological symptoms as well as risk and protective factors. The GPS includes symptoms of PTSD, complex PTSD, anxiety, depression, dissociation, substance abuse, sleep problems, self-harm behaviour and other stress-related problems. The GPS was developed by an international group of experts representing traumatic stress societies worldwide called the “Global Collaboration on Traumatic Stress” [20–26]. The present study aimed to translate, adapt, and examine the psychometric properties of a Persian version of the GPS in Iran.

## Methods

### Part 1 translation content validation and pilot testing

#### Translation and cross-cultural adaptation process

The GPS was translated and adapted based on the seven-step procedure described by Sousa and Rojjanasrirat (2011) for the cross-cultural translation, adaptation and validation of health-related scales [27]. The translation to Persian/Farsi was completed by two independent translators and reviewed by a committee consisting of a psychiatrist, a psychologist, and a professional English translator to check the clarity of the instructions, format of items and responses, and equivalence of content.

A draft of the translated GPS was then sent to six experts, including two psychiatrists with experience in psychotrauma, one mental health expert, one epidemiologist, and two people with a history of psychotrauma as lay experts. They were asked to rate GPS questions on the relevancy, clarity, and comprehensiveness. The content validation process was conducted in two phases [28]. Inter-rater agreement (IRA) was calculated among the experts for the relevance and clarity of each item on the GPS. The item content validity index (I-CVI) for each question was defined as the proportion of experts and lay experts who chose the item as ‘appropriate/clear’ or ‘quite appropriate/clear’. A cutoff of 80% was considered acceptable for this index. The scale content validity index (S-CVI) was also calculated based on the average method (S-CVI/Ave). The acceptable value for S-CVI/Ave was set at 90%. The same procedure was conducted for relevancy of each GPS item. Comprehensiveness of the GPS was assessed by the proportion of experts who reported that the instrument comprehensiveness was appropriate. The acceptable comprehensiveness was set at 80% [29, 30].

#### Pilot testing

Thirty participants (mean age = 29.13; *SD* = 9.16; range = 18–49) were recruited via the University and filled out the questionnaire twice in a two-week interval [31]. Intraclass correlation was used to assess test–retest reliability (< 0.40 = poor; 0.41-0.6 = fair; 0.61-0.80 = moderate; > 0.80 = excellent) and Cronbach’s alpha was calculated to assess internal consistency at baseline.

### Part 2 epidemiological survey

#### Participants and procedure

Using a multistage sampling method, a representative sample of 800 adults from Kermanshah, a province in Eastern Iran, were invited to participate in this cross-sectional study from June 2019 to November 2019. All residents of Kermanshah province experienced a 7.3 magnitude earthquake hit Kermanshah on November 12, 2017 and more than 3000 aftershocks after that. The sample was recruited with the help of Kermanshah Medical Sciences University, Kermanshah, Iran. All residents of 8 municipality areas of Kermanshah aged between 18–65 years, constituted the study reference population. These eight areas are stratified based on socioeconomic status. After selecting three areas randomly from stratified municipal areas in Kermanshah city (primary units), 800 households, proportional to the population size of each selected area, were selected as secondary units. Using the Kish method, one eligible family member (aged 18 years or above) in each household was randomly selected [32]. Four trained researchers visited each household and explained the procedures and goals of the research study. After obtaining written informed consent from willing participants, the researchers provided hard copies of the self-report questionnaires. The completed surveys were retrieved after a week in a closed envelop.

#### Instruments

##### Lifetime traumatic events

Data on the presence of lifetime traumatic events were obtained using the questions; “Have you experienced a specific, stressful event during your life, and what is the worst event?”. Responses include 17 options: a) Natural disaster, b) Fire or explosion, c) Transportation accident, d) Serious accident at work, home, or during recreational activity, e) Exposure to toxic substance, f) Physical assault, g) Assault with a weapon, h) Sexual assault, i) Other unwanted or uncomfortable sexual experience, j) Combat or exposure to a war zone (as soldier or civilian), k) Captivity (e.g., being kidnaped, abducted, held hostage, prisoner of war), l) Life-threatening illness or injury, m) Severe human suffering, n) Sudden violent death (e.g., homicide, suicide), o) Sudden accidental death, p) Serious injury, harm, or death you caused to someone else, and q) Any other very stressful event or experience [33].

##### Global Psychotrauma Screen (GPS)

The GPS was developed to screen for a wide range of trauma-related psychological problems and risk factors and protective factors. The instrument includes 22 items in a yes/no format. The GPS total score is calculated using all 22 items (range 0–22). The total symptom score is calculated by adding up the 17 symptom items (GPS-Sym; range 0–17 with higher scores indicating higher symptom endorsement).

The instrument subdomain scores are calculated by adding up the items for: PTSD (5 items; range 0–5), Disturbances in Self-Organisation (DSO; 2 items; range 0–2), Anxiety (2 items; range 0–2), Depression (2 items; range 0–2), Sleep problems (1 item; range 0–1), Self-harm behaviour (1 item; range 0–1), Dissociation (2 items; range 0–2), Other physical, emotional or social problems (1 item; range 0–1), and Substance abuse (1 item; range 0–1). A Complex PTSD score is the sum of PTSD and DSO items (7 items; range 0–7). A risk factor score is calculated by adding up the 5 risk and protective items (range 0–5). These include: other stressful events (item 17), Childhood trauma (item 19), History of mental illness (item 20) Social support (item 21), Psychological resilience (item 22). The original validation studies in other languages showed a high reliability and good construct validity of the measure [21–25]. The GPS is currently available in over 30 languages and is freely available on https://www.global-psychotrauma.net/gps.

##### PTSD Checklist for DSM-5 (PCL-5)

The PCL-5 is one of the most widely used self-report measures of PTSD [34, 35]. This checklist has an adapted and validated version in Persian [18]. The PCL-5 has 20 items and four subscales, corresponding to the symptoms and clusters of the diagnostic criteria of PTSD in the DSM-5: intrusions, avoidance, negative alterations in cognitions and mood, and hyperarousal.

##### General health questionnaire

The general health questionnaire (GHQ) is a 28-item questionnaire developed by Goldenberg (1972) and translated in Persian by Noorbala, Bagheri, and Mohammad (2009) [36, 37]. This questionnaire includes four subscales: somatic symptoms, anxiety and insomnia, social dysfunction and depression [38]. The authors reported acceptable reliability and validity for this questionnaire [37, 39].

#### Statistical analyses

##### Exploratory and confirmatory factor analysis

The factor structure of the GPS is explored with a tetrachoric exploratory factor analysis (EFA) conducted on a randomly selected subsample of 355 participants (50% of the sample) using the 17 symptom items. The *Kaiser-Meyer- Olkin* > 0.8 along with Bartlett test for sphericity (*p* < 0.05) was used for testing the assumptions of EFA. To confirm the hypothesized factor structure, confirmatory factor analysis (CFA) using maximum likelihood estimation was conducted on the remaining 50% of the sample. The following goodness-of-fit indicators were considered as a guide for acceptable model fit: *Chi-squared/df* < *5*, Root Mean Square Error of Approximation *(RMSEA)* < *0.08*, and *Comparative Fit Index (CFI), Goodness of Fit Index (GFI), Tucker-Lewis index (TLI)* > *0.9*, and standardized root mean squared residual *(SRMR)* < *0.08* [40, 41].

### Reliability and validity

We assessed the reliability of the GPS by investigating inter-item and item-total correlations. We assessed the internal consistency of the GPS total symptom score using Cronbach’s alpha. We assessed the convergent validity between the GPS total symptom score, GHQ and PCL-5 with Pearson’s correlation coefficient.

### Screening accuracy

The accuracy of the GPS for identifying individuals presenting probable PTSD diagnosis was assessed using Receiver Operating Characteristic (ROC) analysis. Individuals were divided into two groups using the recommended PCL-5 cut-off score of 33 [35], to differentiate between those with and without probable PTSD. The Youden index, sensitivity, specificity, and the area under the ROC curve were calculated to estimate the optimal cut-off point for screening accuracy of the GPS total symptom score for probable PTSD.

## Results

### Part 1

#### Reliability and content validity of the GPS: pilot study

Thirty participants (*mean age* = *29.13; SD* = *9.16; range* = *18–49*) participated in this part of the study. The internal consistency of the GPS total symptom score was satisfactory (Cronbach's *α* = 0.73) and the test–retest reliability of the GPS total symptom score was excellent (*ICC* = 0.935 [95% CI: 0.84, 0.97]). Inter-rater agreement between content and lay experts for both relevancy and clarity was high (92%). Based on these results, no modifications were made to the content or wording of the GPS. The I-CVI for relevance and clarity of items ranged between 0.75 and 1 and the S-CVI ranged between 0.95 and 1 (see Additional file 1: Appendix I).

### Part 2

#### Sample characteristics

Of the 800 selected households with eligible participants, 715 (89.40%) agreed to participate in the study. The mean age of participants was 35.72 (*SD* = 10.64), and 49.80% were female-identified. Over one-third (38.18%) of participants had some level of postsecondary education (Table 1).

**Table 1:** Characteristics of study 2 participants by gender (*n* = 715)

**Variables**	**Women (*n* = 356)** **N (%)**	**Men (*n* = 359)** **N (%)**
**Age (years); mean (SD)**	35.01 (9.22)	36.42 (11.84)
**Marital status**		
Single	103 (38.87)	162 (61.13)
Married	227 (54.05)	193 (45.95)
Widow/ Divorced	26 (92.86)	2 (7.14)
**Highest level of education**		
Primary school	101 (60.12)	67 (39.88)
Diploma	128 (47.06)	144 (52.94)
Associate degree	38 (49.35)	39 (50.65)
Bachelor degree	75 (48.70)	79 (51.30)
Master's degree and higher	13 (30.95)	29 (69.05)
**Job Title**		
Housekeeper	214 (100.0)	0 (0)
Self-employed	60 (21.66)	217 (78.34)
Government or private employee	24 (33.33)	48 (66.67)
Retired	5 (16.67)	25 (83.33)
Unemployed	50 (43.48)	65 (56.52)
**History of chronic disease**		
Yes	30 (43.48)	39 (56.52)
No	325 (50.47)	319 (49.53)
**Smoke at least 100 cigarettes in lifetime**		
Yes	20 (11.76)	150 (88.24)
No	336 (61.65)	209 (38.35)
**Current Smoking**		
Yes, daily	11 (11.70)	83 (88.30)
Yes, sometimes	14 (20.59)	54 (79.41)
No	331 (59.86)	222 (40.14)

#### Prevalence of traumatic events

Of the 715 participants, 691 people responded to this question and reported at least one potentially traumatic event. Nearly 33% reported experiencing a natural disaster, 15.48% reported sudden accidental death, 15.05% reported severe human suffering, and 9.41% reported life-threatening illness or injury. Nearly 20% experienced another very stressful event (see Table 2).

**Table 2:** The worst experienced traumatic event in the study sample (*n* = 715)

Trauma Type	N (%)
**Natural disaster (for example, flood, hurricane, tornado, earthquake)**	231 (32.31)
**Transportation accident**	28 (3.92)
**Physical assault (e.g., being attacked, hit, slapped, kicked, beaten up)**	2 (0.28)
**Sexual assault (e.g., rape, attempted rape, made to perform any type of sexual act through force or threat of harm)**	5 (0.70)
**Combat or exposure to a war zone (in the military or as a civilian)**	6 (0.84)
**Captivity (e.g., being kidnaped, abducted, held hostage, prisoner of war)**	2 (0.28)
**Life-threatening illness or injury**	65 (9.09)
**Severe human suffering**	104 (14.55)
**Sudden violent death (e.g., homicide, suicide)**	2 (0.28)
**Sudden accidental death**	107 (14.97)
**Any other very stressful event or experience**	139 (19.44)
**No response to the question**	24 (3.36)

#### GPS, PCL-5 and GHQ scores

**Fig. 1:**
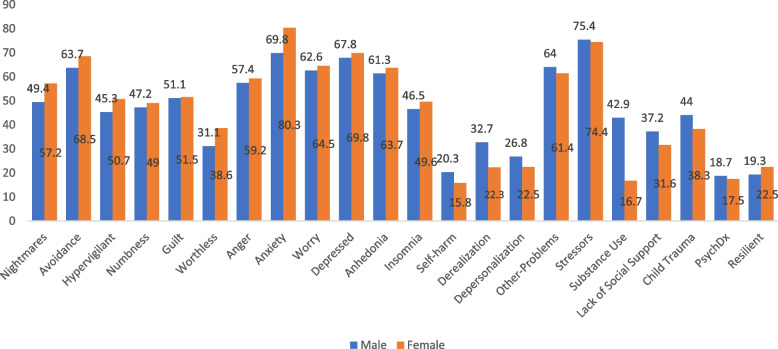
Percent endorsement of GPS items by gender

The mean GPS total score was 10.86 (*SD* = 4.64), the mean GPS-Sym was 8.38 (*SD* = 4.07), and the mean risk and protective factor score 1.88 (*SD* = 1.21). About half (52%) of the participants met criteria for probable PTSD. Individual item endorsement is shown in Fig. 1. Table 3 shows the GPS total and domain scores by gender. No significant gender differences were found for the total scores. Women reported higher scores on the GPS domains of PTSD, DSO, and anxiety compared to men, while men more often endorsed self-harm, dissociation, and substance abuse. Moreover, PTSD domain scores were higher in widowed/divorced participants (71.4%) than in married (53.3%) and single participants (57.4%) (Table 3). The mean PCL-5 score was 29.01 (*SD* = 16.35) and the mean GHQ score was 25.32 (*SD* = 10.56). The GHQ scores were slightly higher in women than men (Table 3).

**Table 3:** Comparison of symptom subscales by gender (*n* = 715)

**Variables**	**Women N (%)**	**Men N (%)**	*P*-value^*^	**Cohen's d Effect size**
**PTSD; Mean (SD)**	2.77 (1.61)	2.56 (1.47)	0.21	-.13
**DSO; Mean (SD)**	0.97 (0.80)	0.88 (0.75)	0.22	-.12
**Complex PTSD; Mean (SD)**	3.73 (2.16)	3.42 (1.94)	0.16	-.14
**Anxiety; Mean (SD)**	1.45 (0.73)	1.32 (0.78)	0.13	-.16
**Depression; Mean (SD)**	1.33 (0.77)	1.29 (0.75)	0.46	-.05
**Sleep Problems (Yes)**	176 (51.46)	166 (48.54)	0.53	-.06
**Self-Harm (Yes)**	56 (43.75)	72 (56.25)	0.22	-.12
**Dissociation; Mean (SD)**	0.44 (0.71)	0.59 (0.77)	0.65	.20
**Substance abuse (Yes)**	59 (27.83)	153 (72.17)	0.013	.60
**Resilience (Yes)**	275 (48.76)	289 (51.24)	0.28	.08
**GPS Total Score; Mean (SD)**	10.78 (4.71)	10.94 (4.57)	0.69	.03
**GPS-Sym; Mean (SD)**	8.39 (4.15)	8.38 (3.99)	0.98	-.001
**RP-Factor; Mean (SD)**	1.84 (1.23)	1.94 (1.18)	0.26	.08
**GHQ; Mean (SD)**	26.12 (10.64)	24.81 (10.16)	0.09	-.13
**PCL; Mean (SD)**	29.90 (16.51)	28.45 (15.97)	0.23	-.09

^*^Corrected for Multiple Comparison

#### Exploratory factor analysis

Kaiser-Olkin-Mayer measure for sampling adequacy (0.85) and Bartlett test of sphericity (*p* < 0.001) confirmed the appropriateness of data for EFA analysis. In the 17 included items, there was no missing data. Tetrachoric EFA indicated a three-factor solution, with most factor loadings ranging between 0.40 and 0.88 (see Table 4) except for item 1 (factor loading 0.28), item 12 (factor loading 0.22), item 13 (factor loading 0.27) and item 17 (factor loading 0.22). The three factors represent: 1. Negative Affect, 2. Core post-traumatic stress symptoms (Core-PTSD), and 3. Dissociative symptoms.

**Table 4:** Item loadings for GPS symptoms

Item	Negative Affect	Dissociation	Core-PTSD
**Sometimes things happen to people that are unusually or especially frightening, horrible, or traumatic. In the past month, have you…**			
had nightmares about the past traumatic life event(s) you have experienced or thought about the event(s) when you did not want to?			0.28
tried hard not to think about past traumatic life event(s) or went out of your way to avoid situations that reminded you of the event(s)?			0.48
been constantly on guard, watchful, or easily startled?			0.50
felt numb or detached from people, activities, or your surroundings?	0.48		
felt guilty or unable to stop blaming yourself or others for past traumatic life event(s) or any problems the event(s) caused?	0.52		
tended to feel worthless?	0.64		
experienced angry outbursts that you could not control?	0.77		
been feeling nervous, anxious, or on edge?	0.88		
been unable to stop or control worrying?	0.74		
been feeling down, depressed, or hopeless?	0.88		
been experiencing little interest or pleasure in doing things?	0.43		
had any problems falling or staying asleep?			0.22
tried to intentionally hurt yourself?	0.27		
perceived or experienced the world or other people differently, so that things seem dreamlike, strange or unreal?		0.40	
felt detached or separated from your body (for example, feeling like you are looking down on yourself from above, or like you are an outside observer of your own body)?		0.55	
had any other physical, emotional or social problems that bothered you?	0.63		
tried to reduce tensions by using alcohol, tobacco, drugs or medication?	0.22		

#### Confirmatory factor analysis

The CFA results for the three-factor solution showed a good fit to the data (χ^2^ = 175.81; *df* = 108; normed χ^2^ = 1.62 < 5; *CFI* = 0.936; *TLI* = 0.920; *SRMR* = 0.051, *RMSEA* = 0.04 (90% *CI*: 0.031 to 0.054) and P-close = 0.84). We also evaluated goodness of fit of the three-factor solution without the items with factor loading < 0.3 (For factor loadings, see supplementary file). The fit statistics indicated minimal improvement (χ2 = 82.86; df = 60; normed χ2 = 1.38 < 5; CFI = 0.969; TLI = 0.960; SRMR = 0.041, RMSEA = 0.03 (90% CI: 0.012 to 0.050) and P-close = 0.952). In addition, the goodness of fit one-factor solution with all items was assessed; again, the fit statistics improved slightly (χ2 = 276.71; df = 185; normed χ2 = 1.49 < 5; CFI = 0.93; TLI = 0.91; SRMR = 0.049, RMSEA = 0.038 (90% CI: 0.029 to 0.047) and P-close = 0.984).

#### Reliability

The item-scale correlation ranged between 0.23 and 0.58. All reported correlations were significant at 0.01 level. In addition, the internal consistency of the GPS total symptom score was excellent (Cronbach's *α* = 0.83; see Table 5).

**Table 5:** Corrected Item–Total Correlations Between Items and GPS-SYM

	**Corrected Item-Total Correlation**
had nightmares about the past traumatic life event(s) you have experienced or thought about the event(s) when you did not want to?	.52
tried hard not to think about past traumatic life event(s) or went out of your way to avoid situations that reminded you of the event(s)?	.29
been constantly on guard, watchful, or easily startled?	.37
felt numb or detached from people, activities, or your surroundings?	.43
felt guilty or unable to stop blaming yourself or others for past traumatic life event(s) or any problems the event(s) caused?	.47
tended to feel worthless?	.49
experienced angry outbursts that you could not control?	.48
been feeling nervous, anxious, or on edge?	.49
been unable to stop or control worrying?	.47
been feeling down, depressed, or hopeless?	.58
been experiencing little interest or pleasure in doing things?	.33
had any problems falling or staying asleep?	.44
tried to intentionally hurt yourself?	.34
perceived or experienced the world or other people differently, so that things seem dreamlike, strange or unreal?	.37
felt detached or separated from your body (e.g., feeling like you are looking down on yourself from above, or like you are an outside observer of your own body)?	.34
had any other physical, emotional or social problems that bothered you?	.42
tried to reduce tensions by using alcohol, tobacco, drugs or medication?	.23

#### Convergent validity

The correlation between GPS-Sym and both PCL-5 (*r* = 0.637, *p* < 0.01) and GHQ *r* = 0.591, *p* < 0.001) was high (Table 6). The correlations between GPS total, and GPS domain scores with PCL-5 and GHQ are shown in Table 6.

**Table 6:** Correlation matrix of convergent validity

	GPS Total	GPS-Sym	RP-Factor	PTSD	DSO	Complex PTSD	Anxiety	Depression	Sleep problems	Self-harm behaviour	Dissociation	Substance abuse	Resilience
Total PCL-5	0.63^***^	0.64^***^	0.38^***^	0.58^***^	0.54^***^	0.61^**^	0.39^***^	0.39^***^	0.33^***^	0.26^***^	0.30^***^	0.15^***^	-0.19^***^
Intrusions	0.44^**^	.45^**^	.25^**^	.45^**^	.35^**^	.47^**^	.28^**^	.26^**^	.28^**^	.16^**^	.17^**^	.02	-.09^*^
Avoidance	0.26^**^	.26^**^	.25^**^	.29^**^	.19^**^	.19^**^	.19^**^	.15^**^	.07	.12^**^	.10^**^	-.01	-.02
Negative Alterations in Cognitions and Mood	0.54^**^	.56^**^	.32^**^	.51^**^	.48^**^	.56^**^	.35^**^	.35^**^	.24^**^	.21^**^	.27^**^	.13^**^	-.19^**^
Hyperarousal	0.52^**^	.52^**^	.34^**^	.45^**^	.42^**^	.49^**^	.32^**^	.32^**^	.28^**^	.22^**^	.26^**^	.14^**^	-.17^**^
Total GHQ	0.56^***^	0.59^***^	0.30^**^	0.50^***^	0.47^***^	0.55^***^	0.36^***^	0.33^***^	0.37^***^	0.31^***^	0.31^***^	0.18^***^	-0.19^***^
Somatic Symptoms	.48^**^	.50^**^	.32^**^	.42^**^	.38^**^	.45^**^	.30^**^	.31^**^	.29^**^	.27^**^	.26^**^	.15^**^	-.21^**^
Anxiety and Insomnia	.63^**^	.66^**^	.34^**^	.57^**^	.55^**^	.63^**^	.43^**^	.37^**^	.44^**^	.38^**^	.29^**^	.17^**^	-.22^**^
Social Dysfunction	-.17^**^	-.19^**^	-.19^**^	-.13^**^	-.19^**^	-.17^**^	-.15^**^	-.12^**^	-.09^*^	-.13^**^	-.02	-.03	.22^**^
Severe Depression	.55^**^	.59^**^	.35^**^	.47^**^	.50^**^	.54^**^	.37^**^	.33^**^	.31^**^	.41^**^	.38^**^	.18^**^	-.32^**^

^*^p < 0.05, ** *p* < 0.01, *** *p* < 0.001

#### Screening accuracy

A GPS total symptom score of 8 was the optimal cut-off for probable PTSD: Youden index = 0.48; sensitivity = 0.87 (95% *CI* 0.83-0.90), specificity = 0.61 (95% *CI* 0.56—0.66), *PPV* = 0.64 (0.59-0.71), *NPV* = 0.86 (0.81—0.88), *DLR* +  = 2.23 (95% CI 1.96 – 2.54) and *DLR-* = 0.21 (0.16—0.29).

**Fig. 2:**
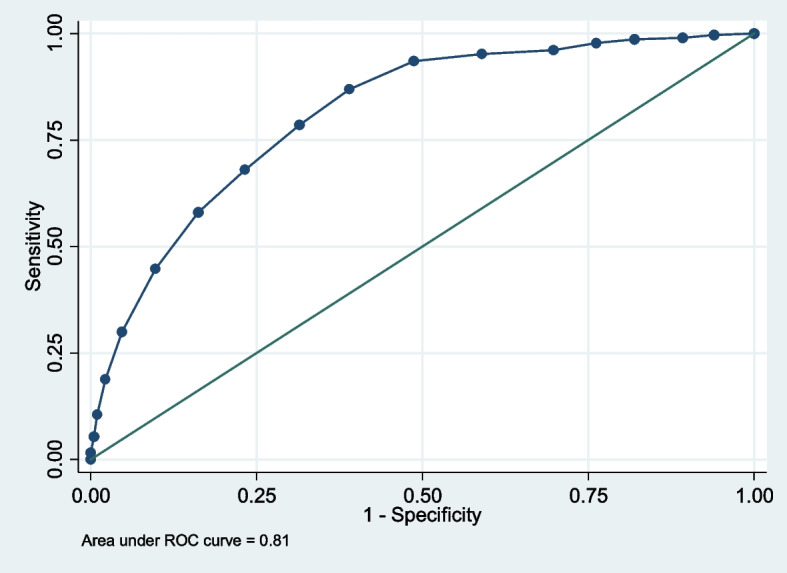
ROC and area under the curve for the GPS symptom score

Maximizing sensitivity for screening purposes a GPS symptom score of 7 would yield a sensitivity 93.6%; for higher specificity a GPS symptom score of 12 yielded specificity 90.2%. The screening accuracies of the GPS symptom scores at various cut-off points are presented in Table 7. The areas under the ROC curve value for GPS symptom scores (0–17) was 0.81 (95% *CI*: 0.78, 0.84) for screening ability (Fig. 2).

**Table 7:** ROC analysis for GPS vs. PCL-5 (with cut-off score of 33)

GPS-Sym Cut-off	Sensitivity	Specificity
	%	%
** > = 0**	100.00%	0.00%
** > = 1**	99.68%	6.00%
** > = 2**	99.04%	10.75%
** > = 3**	98.72%	18.00%
** > = 4**	97.76%	23.75%
** > = 5**	96.17%	30.25%
** > = 6**	95.21%	41.00%
** > = 7**	93.61%	51.25%
** > = 8**	86.90%	61.00%
** > = 9**	78.59%	68.50%
** > = 10**	68.05%	76.75%
** > = 11**	58.15%	83.75%
** > = 12**	44.73%	90.25%
** > = 13**	30.03%	95.25%
** > = 14**	18.85%	97.75%
** > = 15**	10.54%	99.00%
** > = 16**	5.43%	99.50%
** > = 17**	1.60%	100%
** > 17**	0.00%	100%

## Discussion

The present study aimed to adapt and validate a Persian version of the GPS in Kermanshah, a province in Western Iran. The study also provides data from a representative sample in Western Iran on the prevalence of traumatic events and reported trauma-related symptoms.

We first constructed the Persian version of the GPS using forward and backward translations and the Sousa and Rojjanasrirat (2011) seven-step procedure [27]. The items of the translated GPS were deemed relevant and clear based on the pilot testing. Based on a representative sample from Western Iran, we conclude that the GPS is a reliable and valid measure of trauma-related symptoms in Iran. In the current study, the mean total GPS score was 10.86 (4.64) while other samples in Japan, Italy, and across English speaking countries found total GPS scores ranging from 9.1 to 10.92. Scores may be slightly higher in the Iranian sample because of the high prevalence of numerous and ongoing traumatic events including war exposure, economic difficulties, and natural disasters. More research is needed on trauma responses in Eastern countries to compare with the findings of this study.

Previous studies [42–45] indicated substantial gender differences in GPS scores. In most studies, women showed higher levels of trauma-related symptoms compared to men. This may be explained by several factors including the type of trauma (e.g., higher prevalence of sexual trauma among women), age at the time of trauma, the type of coping strategies used (e.g., women use more emotion-focused, defensive and palliative coping), and gender discrimination [43, 46]. In the present study, women scored higher on specific subdomains of PTSD, DSO and anxiety, but men reported higher scores on the dissociation and substance abuse subdomains. On the GPS total symptom score, there was no significant difference between men and women. This might be explained by specific cultural circumstances, for example women having less access to substances.

Exploratory factor analyses and subsequent confirmatory factor analyses produced a three-factor model: (a) Negative Affect; (b) Core-PTSD symptoms, and (c) Dissociative symptoms. Our findings were consistent with the study from Rossi and colleagues (2021), where a three-factor model was identified: (a) Negative Affect; (b) Core-PTSD symptoms, and (c) Dissociative symptoms. In contrast, Frewen et al. (in press) suggests a one-factor model. The distinction between negative affect, core PTSD symptoms and dissociative symptoms is in line with classification systems like the DSM-5 and the International Classification of Diseases – 11th edition (ICD-11) which distinguish affective disorders from PTSD. The DSM-5 also specifies dissociative responses for PTSD (‘PTSD with dissociative symptoms’). Additionally, the distinction between 'Core PTSD' symptoms versus other factors is congruent with the ICD-11 differentiation of PTSD and Complex PTSD.

The Persian GPS demonstrated satisfactory reliability (α = 0.83) between items and scales and high convergent validity between the GPS total symptom score and the GHQ and PCL-5. Using the PCL-5 cutoff score of 33 to indicate probable PTSD, a GPS symptom score of nine was found with optimum sensitivity (78.6%) relative to specificity (68.5%). In our sample, 52.31% (52.4% men; 52.25% women) scored above nine. This high percentage likely reflects the high levels of exposure to traumatic events in the country, including recent exposure to severe earthquakes. In a clinical setting, scoring above nine on the GPS may indicate a need for further assessment and treatment.

This study has several limitations. Firstly, we did not use clinical interviews to make diagnoses in the sample, so we could only evaluate the clinical utility of the GPS based on probable PTSD. Future studies should investigate whether the GPS also accurately predicts clinician-assessed PTSD diagnosis. Secondly, twenty-four respondents did not response to the question about exposure to a traumatic event. Although all those living in Kermanshah province in 2017 experienced a 7.3 magnitude earthquake, which killed 620 and injured 12,386, we cannot be sure these 24 respondents were in Kermanshah during this event. Thirdly, we focused on PTSD diagnosis in this study and did not include measures for other diagnoses such as depression or generalized anxiety disorder. Finally, we included a sample from Kermanshah in Western Iran, which may not generalize to the whole country. However, the large sample size and the use of the multistage stratified sampling method strengthens the interpretations of the study results.

The Persian GPS is a valid and reliable instrument for assessing trauma-related symptomatology in Iran. This brief screener has utility in both epidemiological research and in clinical practice to quickly assess transdiagnostic symptoms related to traumatic events.

## Supplementary Information

**Additional file 1: Appendix I.**

## Data Availability

The datasets generated and/or analysed during the current study are not publicly available due to limitations of ethical approval and data use agreement funder of study but are available from the corresponding author on reasonable request.

## Abbreviations

PTSD: Posttraumatic stress disorder
GPS: Global Psychotrauma Screen
GHQ: General Health Questionnaire
IRA: Inter-rater agreement
I-CVI: Item Content Validity Index
S-CVI: Scale Content Validity Index
DSO: Disturbances in Self-Organization
EFA: Exploratory Factor Analysis
CFA: Confirmatory Factor Analysis
RMSEA: Root Mean Square Error of Approximation
CFI: Comparative Fit Index
GFI: Goodness of Fit Index
TLI: Tucker-Lewis index
SRMR: Standardized Root Mean Squared Residual
ROC: Receiver Operating Characteristic
Core-PTSD: Core Post-Traumatic Stress
