# Supplementary material for: Trauma and its consequences in Iran: cross-cultural adaption and validation of the Global Psychotrauma Screen in a representative sample
Source: BMC Psychiatry. 2023 Jan 25;23:65. doi: 10.1186/s12888-023-04564-8 (PMC9873548; doi:10.1186/s12888-023-04564-8)
Supplement: Supplementary file 1 — Additional file 1: Appendix I. [file 12888_2023_4564_MOESM1_ESM.docx]

**APPENDIX-I**

| **Table 1**. I- CVI for relevancy and clarity for each item and whole scale | | |
| --- | --- | --- |
|  | I-CVI | |
| Items | relevancy | clarity |
| 1. had nightmares about the past traumatic life event(s) … | 1 | 1 |
| 2. tried hard not to think about past traumatic life event(s) … | 1 | 1 |
| 3. been constantly on guard, watchful, or easily startled? | 1 | 1 |
| 4. felt numb or detached from people, activities, or your … | 1 | 1 |
| 5. felt guilty or unable to stop blaming yourself or others … | 1 | 1 |
| 6. tended to feel worthless? | 1 | 1 |
| 7. experienced angry outbursts that you could not control? | 1 | 1 |
| 8. been feeling nervous, anxious, or on edge? | 1 | 1 |
| 9. been unable to stop or control worrying? | 1 | 1 |
| 10. been feeling down, depressed, or hopeless? | 1 | 1 |
| 11. been experiencing little interest or pleasure in doing things? | 1 | 1 |
| 12. had any problems falling or staying asleep? | 1 | 1 |
| 13. tried to intentionally hurt yourself? | 1 | 1 |
| 14. perceived or experienced the world or other people differently… | 1 | 1 |
| 15. felt detached or separated from your body … | 1 | 1 |
| 16. had any other physical, emotional or social problems ... | 1 | 1 |
| 17. experienced other stressful events? | 1 | 1 |
| 18. tried to reduce tensions by using alcohol, tobacco, drugs … | 1 | 1 |
| 19. missed supportive people near you that you could … | 1 | 1 |
| 20. During ***your childhood*** (0-18 years), did you experience … | 1 | 1 |
| 21. Have you ***ever*** received a psychiatric diagnosis … | 1 | 1 |
| 22. Do you ***generally*** consider yourself to be a resilient person? | 0.75 | 1 |
| Ave/S-CVI | 95.48863636 | 1 |
| **Abbreviations:** S-CVI, scale content validity index; I-CVI, item content validity index. | | |

| **Table 2.** Item loadings for GPS symptoms after removing missing traumatic data (n=346). | | | |
| --- | --- | --- | --- |
| Item | Negative Affect | Dissociation | Core-PTSD |
| **Sometimes things happen to people that are unusually or especially frightening, horrible, or traumatic. In the past month, have you….** |  |  |  |
| had nightmares about the past traumatic life event(s) you have experienced or thought about the event(s) when you did not want to? |  |  | 0.30 |
| tried hard not to think about past traumatic life event(s) or went out of your way to avoid situations that reminded you of the event(s)? |  |  | 0.14 |
| been constantly on guard, watchful, or easily startled? |  |  | 0.68 |
| felt numb or detached from people, activities, or your surroundings? | 0.54 |  |  |
| felt guilty or unable to stop blaming yourself or others for past traumatic life event(s) or any problems the event(s) caused? | 0.61 |  |  |
| tended to feel worthless? | 0.60 |  |  |
| experienced angry outbursts that you could not control? | 0.76 |  |  |
| been feeling nervous, anxious, or on edge? | 0.94 |  |  |
| been unable to stop or control worrying? | 0.67 |  |  |
| been feeling down, depressed, or hopeless? | 0.89 |  |  |
| been experiencing little interest or pleasure in doing things? | 0.49 |  |  |
| had any problems falling or staying asleep? |  |  | 0.48 |
| tried to intentionally hurt yourself? | 0.23 |  |  |
| perceived or experienced the world or other people differently, so that things seem dreamlike, strange or unreal? |  | 0.78 |  |
| felt detached or separated from your body (for example, feeling like you are looking down on yourself from above, or like you are an outside observer of your own body)? |  | 0.70 |  |
| had any other physical, emotional or social problems that bothered you? | 0.53 |  |  |
| tried to reduce tensions by using alcohol, tobacco, drugs or medication? | 0.19 |  |  |
